# Local Strain Tuning in Cu Nanoparticles through Glucose-Mediated Synthesis

**DOI:** 10.1021/acsomega.5c03609

**Published:** 2025-10-03

**Authors:** Gustavo Z. Girotto, Kaue G. G. dos Santos, Ruan M. Martins, Marco A. H. Vogt, Silvia Montoro, Fernando Bonetto, Carlos Escudero, André R. Muniz, Fabiano Bernardi

**Affiliations:** † Programa de Pós-Graduação em Física, Instituto de Física, Universidade Federal do Rio Grande do Sul (UFRGS). Av. Bento Gonçalves, 9500, Agronomia, Porto Alegre 91501-970, Brazil; ‡ Departamento de Engenharia Química, Universidade Federal do Rio Grande do Sul (UFRGS). Rua Engenheiro Luiz Englert, s/n°—Prédio 12.204, Farroupilha, Porto Alegre 90040-060, Brazil; § Instituto de Física del Litoral, CONICET-UNL. Guemes 3450, S3000GLN Santa Fe, Argentina; ∥ Institute of Environmental Technology, CEET, VSB—Technical University of Ostrava, 17. listopadu 15/2172, Ostrava-Poruba 70800, Czech Republic; ⊥ ALBA Synchrotron Light Source, Cerdanyola del Vallès, 08290 Barcelona, Spain

## Abstract

Cu nanoparticles are widely used in different fields. Controlling the Cu oxidation state and the local strain is fundamental for optimizing its efficiency in processes, such as catalytic reactions. In this work, Cu nanoparticles were synthesized by using glucose as a reducing agent. Different synthesis conditions led to nanoparticles with a tunable local strain and Cu(0)/Cu_2_O ratio. The amounts of Cu(0) and Cu_2_O are directly related to the local strain in the nanoparticles. The lower amount of Cu(0) gives a longer Cu–Cu distance, and the lower amount of Cu_2_O is associated with longer Cu–O distances. It can be attributed to the creation of interfacial strain at the Cu(0)/Cu_2_O boundaries, as demonstrated by molecular dynamics simulations. Furthermore, the Cu(0) phase is stable at least up to two years in the air due to the presence of gluconate at the surface. This study shows that interfacial strain can be manipulated without the addition of other elements through a facile route.

## Graphical abstract

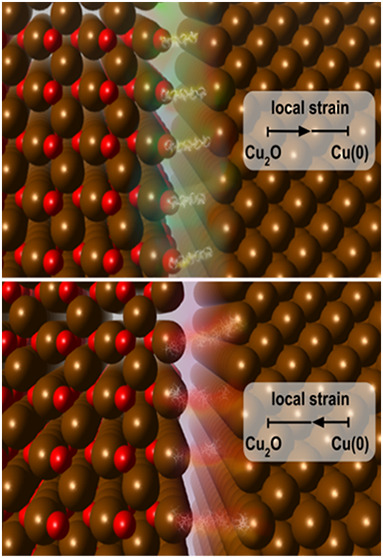

## Introduction

Cu nanomaterials possess properties that make them valuable in a variety of applications, including electronics,^1^ catalysis,^2^ and sensors.^3^ The typical oxidation states, Cu(0), Cu­(I), and Cu­(II), can be exploited for different uses^2,4^ and there are several reports about controlling the Cu oxidation state through synthesis methods.^2^ However, tuning the local strain of Cu nanoparticles is also important but is rarely addressed in the literature. Optimizing the local strain in both Cu_2_O and Cu(0) compounds is the key to enhancing the catalyst’s performance in a broad range of applications. For instance, it can modify the adsorption energies of reaction intermediates of the CO_2_ electroreduction reaction on the Cu(0) catalyst surface, affecting both activity and selectivity.^5,6^ Dendritic Cu presenting nanotwin boundaries has been shown to increase water splitting efficiency.^7^ Lattice tensile strain, which is related to local strain, enhances CO adsorption, thus promoting C–C coupling during CO_2_ reduction reactions, and it results in increased production of C_2+_ products.^5^ Furthermore, tensile strain can decrease the band gap of Cu_2_O.^8^ Therefore, enabling easy local strain tuning in Cu nanoparticles is highly desirable to design new high-performance Cu-based catalysts. Moreover, the oxidation state is linked to the existing local strain, so it is also critical to develop a method that leads to a stable Cu oxidation state over time for a specific application.

Strain can be controlled by synthesis procedures that select the nanoparticle’s overall morphology^9,10^ where surface-induced effects influence the observed strain. Smaller nanoparticles are advantageous for this reason. However, this type of synthesis often requires surfactants to stabilize small particles^10^ or the deposition of nanoparticles on porous structures.^11^ This adds to the general complexity of the synthesis procedure and may eventually lead to undesirable components that remain attached to the Cu nanoparticles. Precise doping can also modify strain,^12^ but diffusion may lead to dopant clustering and bring forth other complications under working conditions.^13^

Cu nanoparticles can be produced from Cu salts following an established reduction method through a combination of NaOH and glucose.^14^ The synthesis methods to obtain Cu(0) usually rely on the use of capping agents or stabilizers, such as polyvinylpyrrolidone (PVP), cetyltrimethylammonium bromide (CTAB), sodium dodecyl sulfate (SDS), and poly­(ethylene glycol) (PEG).^14,15^ However, capping agents may hinder access to the active sites at the surface of the Cu nanoparticles and usually need to be removed through thermal treatment. In this process, some of the interesting properties of the synthesized nanoparticles (size, oxidation state, etc.) are lost. The production of stable nanoparticles without additional capping agents is a great challenge. The copper salt used also influences the kinetics of Cu nanoparticle formation and its resulting morphology.^16,17^ Usually, different morphologies can be obtained under different pH conditions, such as spheres, octahedrons, and hexapods.^18^

In this study, Cu nanoparticles are obtained without using additional capping agents following the NaOH and glucose route. The results obtained demonstrate that even without additional capping agents, it is still possible to reach a highly stable Cu(0) phase in the nanoparticles by varying the synthesis steps used. Additionally, it is also possible to tune the local strain of the Cu(0) and Cu_2_O phases with this method. Molecular dynamics simulations were carried out to analyze in more detail the interaction between Cu(0) and Cu_2_O phases, thus providing additional insights into the local strain at the interfaces.

## Results and Discussion

Figure 1 shows the Fourier-transform infrared (FTIR) measurements during the synthesis process of Cu nanoparticles for two extreme synthesis parameters used, namely, Cu A and Cu H samples (see Table 1). Both samples show the same main trend where the oscillation at around 1020 cm^–1^ is replaced by an oscillation at around 1070 cm^–1^ during the synthesis procedure. These oscillations refer to the C–O stretching vibration from primary alcohols.^19^ The replacement is related to the transformation of glucose to gluconic acid, in accordance with the literature.^20–22^ Indeed, the following reactions can be considered during the synthesis process. Cu^2+^ is dissolved in water according to the equilibrium conditions^23^

1 $${\mathrm{Cu}}^{2+}+H_{2}O⇌{\mathrm{Cu}\left(\mathrm{OH}\right)}^{+}+H^{+}$$

$${\mathrm{Cu}\left(\mathrm{OH}\right)}^{+}+H_{2}O⇌\mathrm{Cu}(O{H)}_{2}+H^{+}$$ 2

**Figure 1:**
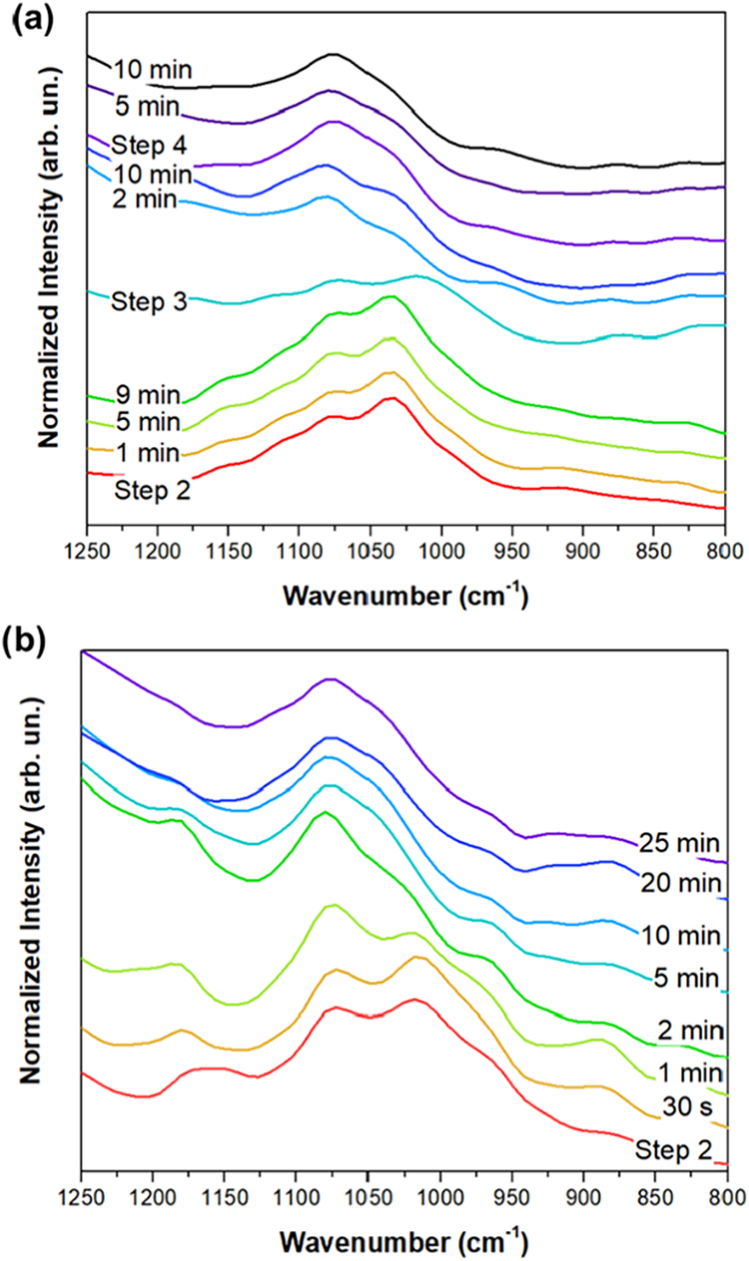
FTIR measurements of the solution during the synthesis of (a) Cu A and (b) Cu H samples.

**Table 1:** Parameters Used in the Synthesis Procedure for the Different Cu Nanoparticles

sample	step 1	step 2	step 3	step 4
Cu A	0.25 M NaOH	0.1 M CuCl_2_·2H_2_O	0.75 M NaOH	0.15 M glucose
	3 mL	0.1 M glucose	1 mL	1 mL H_2_O
		3 mL H_2_O		
Cu B	0.25 M NaOH	0.1 M CuCl_2_·2H_2_O	0.75 M NaOH	0.1 M glucose
	3 mL	0.1 M glucose	1 mL	1 mL H_2_O
		3 mL H_2_O		
Cu C	0.25 M NaOH	0.1 M CuCl_2_·2H_2_O	0.75 M NaOH	0.2 M glucose
	3 mL	0.1 M glucose	1 mL	1 mL H_2_O
		3 mL H_2_O		
Cu D	0.2 M NaOH	0.1 M CuCl_2_·2H_2_O	0.75 M NaOH	0.1 M glucose
	3 mL	0.1 M glucose	1 mL	1 mL H_2_O
		3 mL H_2_O		
Cu E	0.2 M NaOH	0.1 M CuCl_2_·2H_2_O	0.9 M	0.2 M glucose
	3 mL	0.1 M glucose	NaOH	1 mL H_2_O
		3 mL H_2_O	1 mL	
Cu F	0.15 M NaOH	0.1 M CuCl_2_·2H_2_O	0.75 M NaOH	0.25 M glucose 1 mL H_2_O
	3 mL	0.1 M glucose	1 mL	
		3 mL H_2_O		
Cu G	0.1 M CuCl_2_·2H_2_O	0.45 M NaOH		
	0.1 M glucose	3 mL		
	3 mL H_2_O			
Cu H	0.6 M NaOH	0.1 M CuCl_2_·2H_2_O		
	3 mL	0.1 M glucose		
		3 mL H_2_O		

NaOH induces the precipitation of a Cu­(OH)_2_ compound (blue solution) through the change in pH for basic solutions (pH > 8).^23^ With the addition of glucose in step 2 (explained in the MethodsSection), there is a transition of the solution color from green to yellow and finally to red, which corresponds to the growth of Cu_2_O crystals. With the further addition of NaOH in step 3, a brownish precipitate is observed. Cupric hydroxide can be decomposed into CuO during heating. If no glucose is added to the reaction, there is the formation of a black precipitate at the end of the synthesis, following the equation

$${2\mathrm{Cu}\left(\mathrm{OH}\right)}_{2}\left(s\right)\to 2\mathrm{CuO}\left(s\right)+{2H}_{2}O$$ 3

These observations can be proven through X-ray diffraction (XRD) analysis. Figure S1 of the SI shows the XRD pattern of the Cu A sample at the beginning of step 2 of the synthesis procedure. It shows the presence of Cu­(OH)_2_, as expected, together with CuO and NaCl. CuO comes from the drying process in a vacuum of the sample before XRD measurement, since Cu­(OH)_2_ is unstable and easily decomposes into CuO and H_2_O. The NaCl comes from the reaction of NaOH with the Cu salt. Furthermore, it also shows the XRD pattern at the same step but without adding glucose, where only CuO is observed, as expected.

Glucose interaction with transition metals and NaOH can lead to the production of different organic molecules. For example, the presence of Ni^2+^ ions has been shown to promote lactic acid formation.^24^ Cu ions are able to promote the transformation to fructose under pH values close to 5.^23^ Furthermore, because of diffused O, glucose can also be oxidized in alkaline conditions in the presence of Cu^2+^,^25^ which is reduced to Cu^+^. Another widely known example of glucose oxidation is employing Benedict’s reagent (Cu­(II) complex with citrate anions), leading to the formation of carboxylic salts or diketones.^26^

Na^+^ and Cl^–^ concentrations are substantially below the expected saturation point; therefore, the formation of NaCl crystals is negligible. Glucose dissolved in aqueous conditions is a mixture of both α-d-glucopyranose and β-d-glucopyranose anomers, which exist in equilibrium in the process known as mutarotation. It has been reported that the presence of dissolved NaCl does not significantly alter the relative α-d-glucopyranose to β-d-glucopyranose concentration,^27^ but the α-anomer is known to present a higher affinity to complexing with Na^+^ ions.^27^ The acyclic form of glucose exists in a proportion of less than 1% of the solution because the pyranosidic form is preferred.^28^ Also, it is well-known that in alkaline conditions, dextrose is converted via the Lobry de Bruyn–van Ekenstein rearrangement to D-fructose and D-mannose.^29^ Finally, hydrolysis of glucose in alkaline media can lead to shorter molecules.^30^

It is interesting to note that the gradual change in the FTIR spectrum during the synthesis of the Cu H sample indicates that the evolution could be linked to the growth of the Cu_2_O crystal. The FTIR spectra of the Cu A sample show that the further addition of NaOH in step 3 results in an FTIR spectrum closer to the initial one of the Cu H sample. This is also related to the further reduction of Cu_2_O to Cu(0). In this case, the reduction mechanism of Cu^2+^ to Cu_2_O could happen through the following half reactions^30^

4 $$C_{6}H_{12}O_{6}+2{\mathrm{OH}}^{-}\to C_{6}H_{12}O_{7}+H_{2}O+2e^{-}$$

5 $$2{\mathrm{Cu}}^{2+}+2{\mathrm{OH}}^{-}+2e^{-}\to {\mathrm{Cu}}_{2}O+H_{2}O$$

It is also interesting to notice that OH^–^ ions are expected to remain adsorbed on the particles, contributing to the reduction in their aggregation.^31^ Contrary to other studies,^14^ it is expected that the reduction to Cu(0) could happen directly on Cu^+^ sites of the Cu_2_O crystals by the overall reaction^32^

$${\mathrm{Cu}}^{+}+C_{6}H_{12}O_{6}+{3\mathrm{OH}}^{-}\to C_{6}H_{11}O_{7}^{-2}+{2H}_{2}O+\mathrm{Cu}\left(0\right)$$ 6

Figure 2a presents a comparison between the XRD measurements of the Cu nanoparticles synthesized, and Figure 2b shows a close look at the two main Bragg reflections. All samples present a mixture of Cu(0) (ICSD 7954) and Cu_2_O (ICSD 172174) phases. The Cu A sample contains the highest concentration of the Cu(0) phase, while the Cu F, Cu G, and Cu H samples have the highest concentration of the Cu_2_O phase. Moving from sample A to H, the changes are related to when and how much glucose and NaOH are added to the redox reactions. Earlier or larger glucose doses and milder basic conditions drive reduction to metallic Cu(0), while limited or delayed glucose, together with conditions that favor Cu­(OH)_2_ precipitation, promotes the formation of Cu_2_O. The ratio between Cu(0) and Cu_2_O phases can be easily adjusted by modifying the pH during synthesis by varying the NaOH and glucose concentrations. Notably, the Cu_2_O phase concentration becomes significant from sample D onward. Moreover, it is possible to observe that the main Bragg reflection of the Cu_2_O phase at around 36.4° in Cu C, Cu B, and Cu A samples is displaced relatively to the same reflection in the Cu H sample. This is an indicative of size or strain modification. The crystallite sizes of the Cu(0) and Cu_2_O phases were estimated using the Scherrer equation, as presented in Table S1 of the SI, with the Cu(0) phase showing a consistent average size of approximately 30 nm and the Cu_2_O phase exhibiting a variable size range between 20 and 100 nm among the different samples.

**Figure 2:**
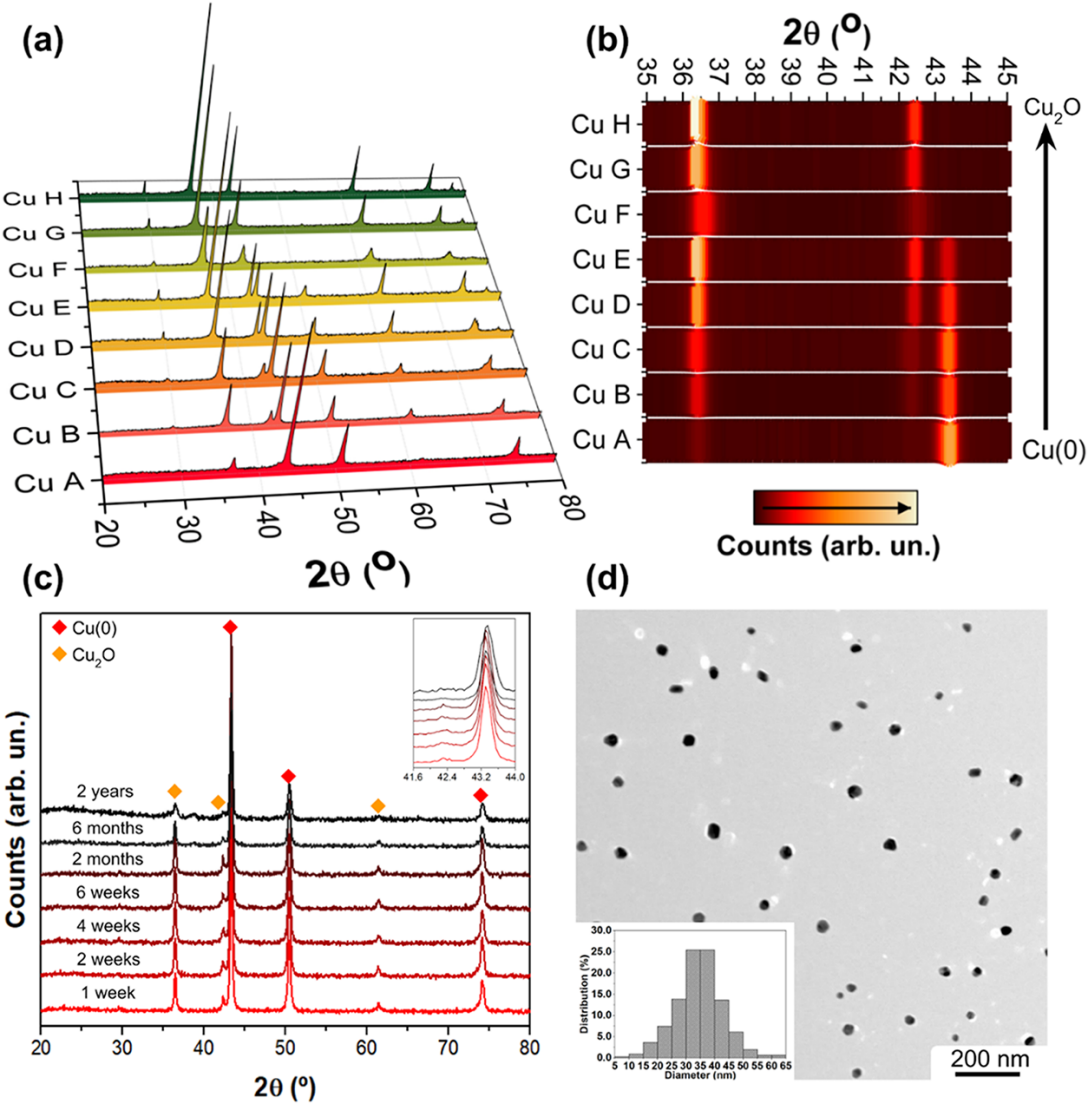
(a) XRD patterns of Cu nanoparticles and (b) zoom between 35 and 45° of the XRD patterns, showing the main Bragg reflections of the cubic Cu_2_O and fcc Cu(0) phases. (c) XRD patterns of the Cu A sample measured at different time periods after the synthesis. (d) Typical transmission electron microscopy (TEM) image of the Cu A sample and the corresponding histogram of size distribution.

Cu­(0) typically oxidizes quickly under air exposure.^33^ Consequently, the ability to optimize and keep a specific Cu(0)/Cu_2_O ratio over time can be significantly affected by aging effects. For this reason, the Cu A sample was selected to investigate this effect, as its high Cu(0) content allows for easier tracking of O content changes. Figure 2c displays the XRD patterns of the Cu A sample measured at different time periods after the synthesis. No noticeable changes were observed for at least up to 2 years, demonstrating the excellent stability of the synthesized samples. It occurs due to the presence of gluconic acid around the nanoparticles, since it has been observed that carboxylic acids are very efficient in preventing air oxidation of Cu.^34^ Indeed, the FTIR spectra of the powder samples were taken after the synthesis and washing procedure, as shown in Figure S2 of the SI. The FTIR spectrum is quite similar to that observed during the synthesis procedure in all samples, thus indicating that the organic molecules are bound to the surface of the nanoparticles. The FTIR spectrum of the Cu A sample after washing with acetone for 30 min, centrifuging, and drying in a vacuum is also shown in the same figure. The feature at 1070 cm^–1^ disappears, indicating the easy removal of the organic molecules from the surface. The Cu A nanoparticles also show a constant Cu(0) crystallite size from the Scherrer equation over time, further confirming the nanoparticles’ stability to aging. Figure 2d presents a typical TEM image of the Cu A sample, which is composed of almost spherical nanoparticles with an average size of 35 ± 5 nm (Figure 2d), which is similar to the Cu_2_O and Cu(0) particle sizes found with the Scherrer equation (Table S1). Even considering the well-known discrepancy in the size obtained from XRD and TEM analysis,^35^ the result in this case shows that both techniques give similar results.

It is important to highlight that gluconic acid prevents Cu oxidation, but it does not form a rigid capping layer that hinders the Cu surface to the gaseous atmosphere in a typical catalytic reaction. It was verified through in situ time-resolved XRD measurements of the Cu A nanoparticles during heating to 200 °C in an oxidizing atmosphere. The XRD patterns of Figure S3 of the SI demonstrate that Cu A sample starts to oxidize from Cu(0) to Cu_2_O at around 180 °C. It means that the presence of gluconic acid does not avoid the use of these nanoparticles in catalysis, even for low-temperature reactions, since the surface is able to react with the gaseous atmosphere. The gluconate ion thickness was estimated from the fitting of the long-scan XPS spectrum of Cu A nanoparticles with the SESSA software (see Figure S4 of the SI). The best fit was found for a 30 nm Cu nanoparticle with a C_15_O_5_ shell of around 1.2 g/cm^3^ density and 3.5 nm thickness. This is the maximum thickness since different organic components can be adsorbed in both the Si substrate and Cu nanoparticles. For catalytic applications, the gluconate ion layer may be easily removed by a washing procedure (see Figure S2) without the need for high-temperature treatments in the sample.

The oxidation state of Cu at the nanoparticle surface is crucial for its catalytic activity. It means the aging effects should be evaluated for the nanoparticle surface as well. XPS measurements were conducted to examine the Cu A nanoparticles right after the synthesis and two months and two years later, as shown in Figure 3a. Initially, the Cu 2p XPS spectrum displays a broad peak at 934.5 eV and a satellite characteristic of the CuO phase.^36^ The other component, consistent with Cu_2_O, contributes approximately 33% of the total peak area. This component is attributed to Cu_2_O, since the FWHM value agrees with that of the Cu_2_O standard, which is slightly wider than that of the Cu(0) standard. After two months, the CuO contribution slightly increases from 67 to 79% but it is stabilized after two years. Observations in the Cu LMM Auger region support this (Figure 3b), with the spectrum initially matching CuO and slightly increasing the shoulder near 916 eV after two months, which stabilizes after two years. The XPS and Auger spectra show a relatively slow oxidation of Cu, even at the surface of the nanoparticles, as compared to the literature where the surface after synthesis is oxidized to CuO even when adding capping layers.^37^ Moreover, it is important to point out that catalysts generally are activated before the catalytic reaction, thus reducing the surface to the main oxidation state of the full nanoparticle.^38^

**Figure 3:**
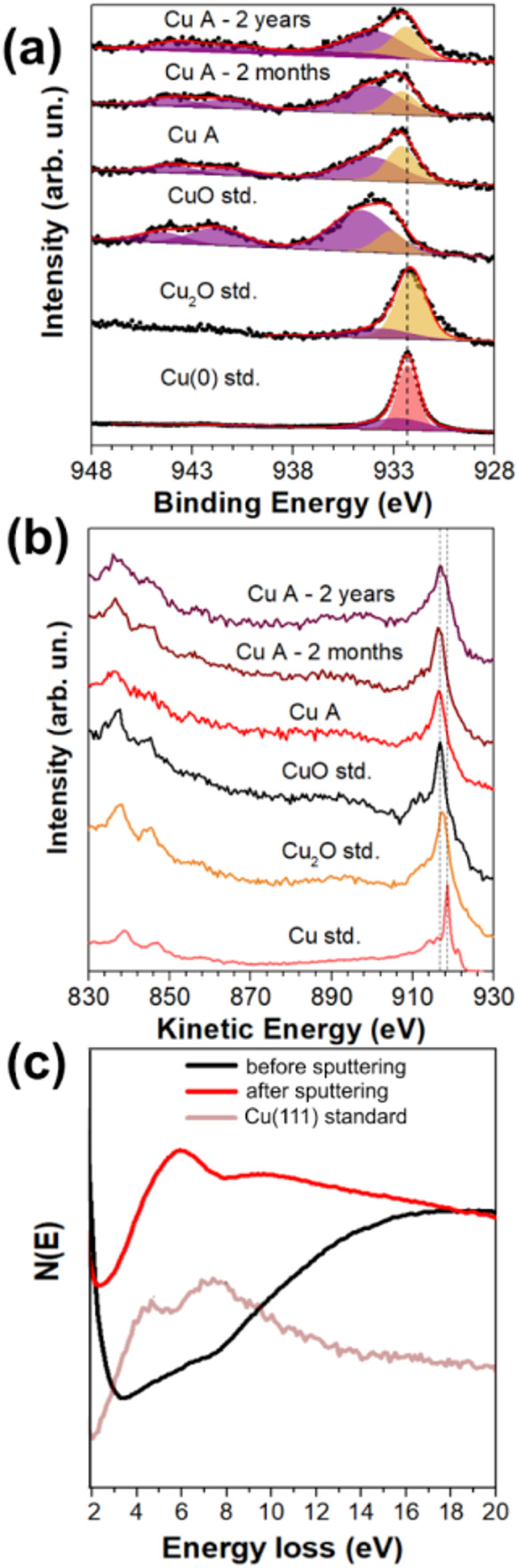
(a) Cu 2p XPS and (b) Cu LMM Auger spectra of Cu A sample synthesized and after 2 months and 2 years from the synthesis, along with Cu(0), Cu_2_O, and CuO standards. (c) REELS spectra of the aged Cu A sample before and after the sputtering procedure.

The REELS spectra are shown in Figure 3c. The REELS spectrum of the Cu(111) standard shows two features at ∼4.3 and ∼7 eV assigned to the excitation of d electrons to states above the Fermi level.^39^ The aged Cu A sample (before sputtering) shows a REELS spectrum with a feature at ∼20 eV coming from C π + σ plasmons.^40^ After sputtering, it is possible to observe an O 2p transition at ∼6 eV coming from CuO or Cu_2_O at the surface.^41^ Indeed, HRTEM measurements prove the presence of an oxide layer at the surface and Cu(0) in the inner part of the nanoparticles, as shown in Figure S5. Thus, it can be argued that since gluconate is present at the Cu surface, it is removed after the sputtering procedure and bound to either Cu^+^ or Cu^2+^ sites, not Cu(0), since the Cu(0) features do not appear in the REELS spectrum after sputtering. It means that the Cu(0) phase is still present as observed in XRD measurements, but not at the surface, as expected.

Figure 4a presents the EXAFS oscillations, and Figure 4 b presents the corresponding FT. The EXAFS oscillations show distinct features from sample A (highest Cu(0)/Cu_2_O ratio) to sample H (lowest Cu(0)/Cu_2_O ratio). With increased oxidation, a damping effect is observed around 8 Å^–1^, while a new oscillation appears near 4.5 Å^–1^, and the doublet at 2.5 Å^–1^ becomes more evident. The FT shows a decrease in Cu–Cu scattering intensity from Cu(0), while the Cu–O scattering intensity from the Cu_2_O phase increases from sample A to sample H, thus aligning with the XRD data trends. It is interesting to notice that the Cu–O scattering distance from the first shell is visibly shifted to low R values with the increase in the Cu_2_O fraction.

**Figure 4:**
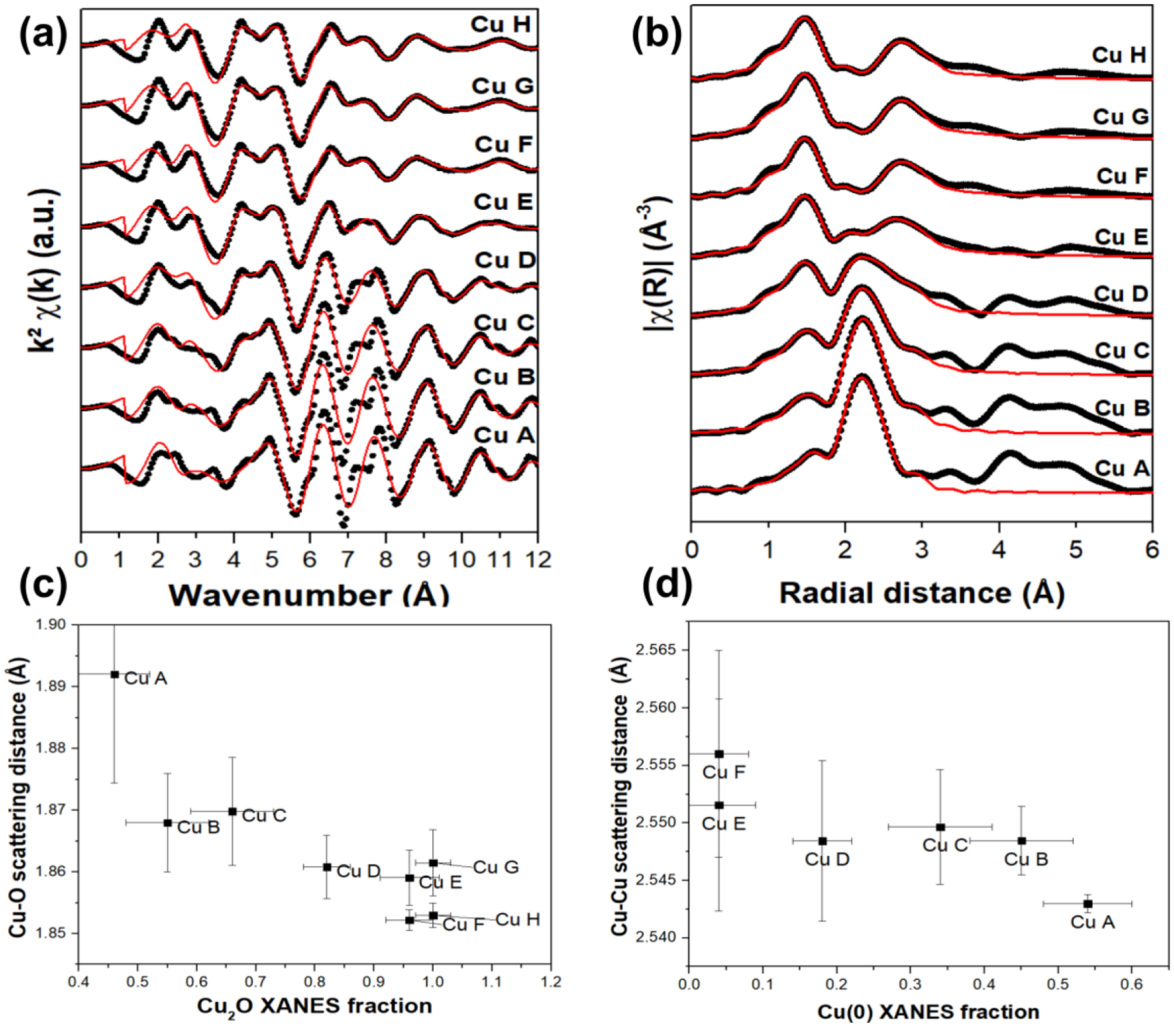
(a) EXAFS oscillations and (b) the corresponding FT. Data are represented as black points and their fitting result as a red line. (c) Cu–O scattering path distance from Cu_2_O phase as a function of the Cu_2_O fraction and (d) Cu–Cu scattering path distance as a function of the Cu(0) fraction, both obtained from the fitting of the EXAFS data.

The parameters obtained from the fit of FT are shown in Table S2 of the SI. Some scattering distances could be correlated to Cu(0) and Cu_2_O fractions obtained using the linear combination analysis of XANES data. The XANES regions are shown in Figure S6 of the SI, along with the fitting result for the Cu A sample as a typical result. Figure 4c shows that the lower the Cu_2_O fraction, the longer the Cu–O distance. Similarly, Figure 4d shows that the Cu–Cu distance for the Cu(0) phase increases for smaller Cu(0) contents. The change in the Cu–Cu and Cu–O distances is directly related to the local strain of the samples. Local strain has been recently calculated for different Cu_2_O and Cu(0) interfaces, where the interface with the minimum local strain is found for the [111]/[111] combination with a value around 2%.^42^ This is almost exactly the same value found for sample Cu A if calculated from the Cu–Cu and Cu–O distance values. The strain observed is consistent with that obtained from the wavelet transform analysis of the HRTEM image of Cu A sample shown in Figure S7 of the SI. Another recent study found that Cu_2_O films over Cu surfaces show undulations to satisfy the matching conditions at the interface.^43^ Interestingly, it was observed that dislocations between Cu_2_O and Cu interfaces tend to reduce the local tensile strain, promoting stability to further oxidation.^44^

Molecular dynamics simulations were carried out to gather a further understanding of the strain at the Cu(0)/Cu_2_O interface. The atomic configurations are illustrated in Figures 5a,b and S1 of the SI (initial states), consisting of a Cu_2_O slab with periodic boundary conditions along the plane (5.15 × 5.15 nm^2^) and hemispheric Cu(0) nanoparticles of varied sizes (with diameters of 1.5, 1.8, and 2.2 nm) deposited on top. For the sake of comparison, the bulk phases of Cu(0) and Cu_2_O were analyzed as well, with computed lattice parameters of 3.67 and 4.29 Å, respectively, consistent with experimental values.

**Figure 5:**
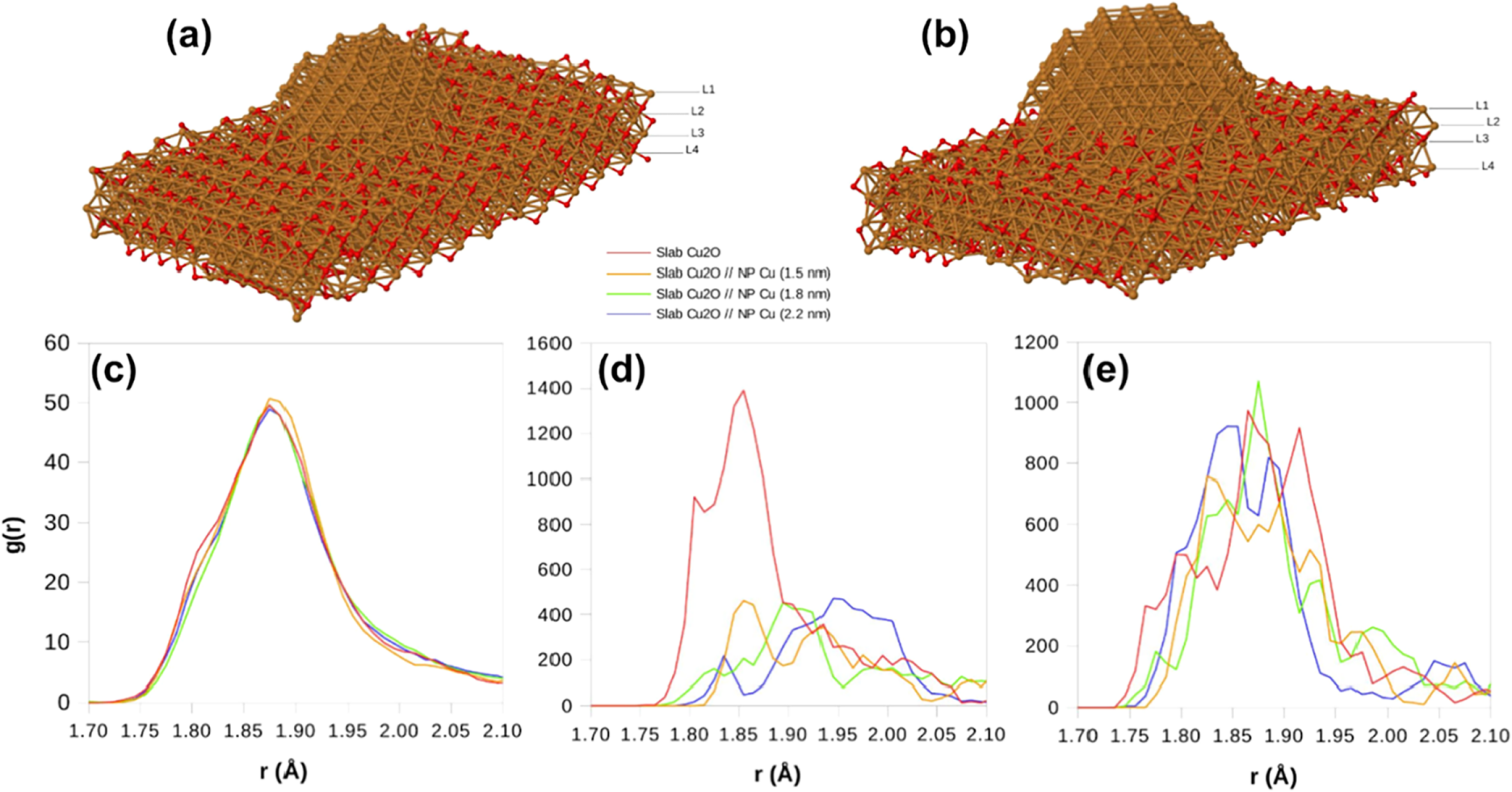
(a, b) Atomic configurations of the Cu/Cu_2_O interfaces analyzed in molecular dynamics simulations consisting of Cu nanoparticles (1.8 and 2.2 nm, respectively) deposited on a Cu_2_O slab. The first peak of the RDF based on the Cu–O interatomic distances is shown for (c) full systems and (d, e) the first (L1) and second (L2) layers of the Cu_2_O substrate, as indicated in (a,b). The RDF for a pristine slab is also shown in (c–e) to assess the comparisons.

Figure 5c–e shows the first peak of the RDFs based on the Cu–O interatomic distances of the different systems analyzed and the full RDFs are shown in Figures S8 and S9 of the SI. The isolated Cu_2_O slab shows a broad first peak due to the presence of surfaces and small slab thickness. The inclusion of Cu(0) nanoparticles at the surface does not significantly change the RDF, where slight deviations are observed at distances far from the peak maximum. These deviations are small due to the relatively lower number of atoms at the interfaces compared to the total number of atoms in the system. To analyze in more detail the origin of these differences, the RDFs were also computed for four individual layers of the substrate (L1–L4, as depicted in Figure 5a,b). The results for the first and second layers (from top to bottom) are shown in Figures 5d,e, while S5, and S6 of the SI show the same for the other layers. While the RDFs of the L2 layer (Figure 5e) for the three systems are relatively similar (the same can be seen for L3 and L4 in Figures S5 and S6 of SI), significant changes are observed for the first layer, L1. The contact of the Cu_2_O slab with the Cu(0) nanoparticles promotes an increase in the Cu–O distances as compared to the pristine slab because the maximum of the RDFs is shifted to higher values. This effect is more significant for larger sizes of the Cu(0) domain, in agreement with the EXAFS result shown in Figure 4a. No significant differences in bond straining are seen for the layers far from the top surface.

Taking into consideration the following analysis, it is reasonable to identify that the formation of the Cu(0) phase after the reduction of Cu_2_O creates regions inside the nanoparticles where local strain at the Cu(0)/Cu_2_O interface is present. One possibility is that the reduction from Cu_2_O to Cu(0) inside a nanoparticle takes place from one side to the other of the grain, together with the formation of Cu(0) islands.^45^ It is reported in the literature that the Cu(0) island formation occurs to minimize the interfacial size during the reduction process using the H_2_ gas.^45^ From the results of MD simulations, it is possible to identify that strain is found to be located mostly at the first Cu(0)/Cu_2_O interface layer. Because XAS is probing the entire nanoparticle, the observation of this strain indicates that there is the formation of a large interfacial area between the Cu(0) and Cu_2_O domains. Since Cu_2_O species are located at the surface, Cu(0) domains must be located inside the nanoparticle. A large Cu(0)/Cu_2_O interfacial area without the addition of further known capping agents, such as PVP or CTAB, and without the addition of other metals can be exploited in different applications. The modulation of the interface plays an important role during CO_2_ reduction to methanol, where it balances the binding energies of the adsorbed species,^46^ and it has been observed that it can even support ferromagnetism.^47^ In this way, the easy synthesis of Cu nanoparticles reported allows local strain tuning, with promising applications in different fields.

## Conclusions

Cu nanoparticles were synthesized by varying the glucose reduction method’s reaction steps. The results point to easy control of the Cu oxidation state and local strain through the synthesis conditions. As observed with EXAFS analysis, the lower the amount of Cu(0) (Cu_2_O), the longer the Cu–Cu (Cu–O) distance of the Cu(0) (Cu_2_O) phase. This local strain is directly linked to the Cu(0)/Cu_2_O interfacial strain, as observed in molecular dynamics simulations, concentrated at the first interfacial layers. Furthermore, gluconate ions are present at the Cu nanoparticle surface, providing long-term stability in the air, in addition to not completely hindering the Cu surface to a gas atmosphere. The gluconate ions can be removed by a simple washing procedure such as dispersion in acetone. The stable Cu nanoparticles can be exploited in different applications that require Cu(0) such as catalysis.

## Methods

CuCl_2_·2H_2_O (Sigma, 99.99%, molar mass 170.5 g/mol, product code 467 847) and anhydrous glucose/dextrose (Cetus Inc., molar mass 180.2 g/mol) were used for the synthesis procedure without further treatment. In a typical synthesis, 3 mL of 0.25 M NaOH was left under an 80 °C thermal bath with magnetic stirring at 270 rpm (step 1). After stirring for 2 min, 3 mL of a 0.1 M CuCl_2_·2H_2_O and 0.1 M glucose solution, with a pH of 5, were added to the alkaline solution, resulting in a pH of 8 of the solution (step 2). After an additional 10 min of stirring, 1 mL of a 0.75 M NaOH solution was added (step 3), with the pH rising to around 12. Finally, after 12 min or more, 1 mL of 0.15 M glucose was mixed with the solution (step 4), and the system was left to react for 25 min. The system was cooled to room temperature, and the powder was centrifuged at 3600 rpm for 10 min, washed with Mili-Q water, and centrifuged again. Finally, the sample was dried under a vacuum inside a desiccator. This procedure leads to Cu nanoparticles that will be labeled as Cu A. The parameters from step 1 to step 4 were modified, thus producing new samples as specified in Table 1.

FTIR measurements of the Cu solution at selected times of the synthesis procedure were performed at the Brazilian Synchrotron Light Source (LNLS). The measurements were done with a Cary 620 (Agilent Technologies) using ATR mode in the mid-IR range (4000–400 cm^–1^) using IR illumination from a Globar source and a MCT detector (mercury–cadmium–telluride). Each spectrum was acquired from 64 averages with a 16 cm^–1^ spectral resolution. The FTIR spectrum of H_2_O was used for background removal. For the measurements, 100 μL were deposited on top of the ATR crystal.

XRD measurements were obtained for the Cu nanoparticles at the Centro de Nanociência e Nanotecnologia (CNANO-UFRGS) in a Rigaku diffractometer with a Cu Kα X-ray source (wavelength of 1.5406 Å), operating at 40 kV and 17 mA with a graphite monochromator. A step size of 0.05° and a scan rate of 0.33°/min were applied during data collection. The XRD pattern analysis and indexing were conducted with PCPPDFWIN version 2.1, utilizing the JCPDS-ICDD database for reference. The crystallite size was determined by using the Scherrer equation by fitting the Bragg reflections with a pseudo-Voigt function.

TEM of the Cu A nanoparticles was carried out at the Centro de Microscopia and Microanálise Brasil-Sul (CMM BR-Sul-UFRGS), using the Jeol JEM 1400 Flash microscope operating at 120 kV. High-resolution transmission electron microscopy (HRTEM) was conducted at the Centro de Microscopia at UFMG (CM-UFMG) with a Tecnai G2–20 SuperTwin FEI microscope operating at 200 kV. The powder was dispersed in Milli-Q water, followed by 1 min of sonication and mechanical stirring. Three 10 μL drops of the solution were then deposited successively onto a Ni grid coated with a carbon film. Image analysis was performed using the ImageJ software. Size distributions were obtained by manually drawing two perpendicular lines across each nanoparticle and averaging the distances.

In situ time-resolved XRD measurements were conducted at the Paineira beamline at the Brazilian Synchrotron Light Source (LNLS). Around 10 mg of the Cu A nanoparticle powder was inserted in a quartz capillary of 1.2 mm diameter in a region 6.0 mm in length. The capillary was fixed in the existing cell at the beamline. The measurements were performed in the Bragg–Brentano geometry (θ–2θ) with an incident monochromatic X-ray beam of *h*ν = 24.5 keV (λ = 0.5061 Å) in the 2θ range from 1 to 109° and a step size of 0.0035°. The X-ray beam was monochromatized with a Si(311) double-crystal monochromator, and the PiMega detector was used. The sample was exposed to 10 mL/min of a 20% O_2_ + 80% N_2_ atmosphere and heated to 200 °C with a 5 °C/min rate. The XRD patterns were collected every 1 min during the full process. The XRD pattern analysis and indexing were conducted with PCPPDFWIN version 2.1, utilizing the JCPDS-ICDD database for reference.

X-ray photoelectron spectroscopy (XPS) measurements of the Cu nanoparticles were performed at the Laboratório Multiusuário de Análise de Superfcies (LAMAS-UFRGS) using an Omicron SPHERA analyzer equipped with an Al Kα X-ray source (*h*ν = 1486.7 eV). The nanoparticle powder was spread over carbon tape for analysis. The base pressure during the measurements was maintained at 10^–8^ mbar. The measurements were taken in the long scan, Cu 2p, Cu LMM, O 1s, and C 1s regions. A pass energy of 50 and 10 eV and energy steps of 1 and 0.05 eV with a dwell time of 0.2 s were used for the long-scan and high-resolution regions, respectively.

The XPS spectra were calibrated using the adventitious carbon position at 284.5 eV. The data were processed with XPS Peak 4.1 software using a Shirley-type background and a Gaussian–Lorentzian sum function (18% Lorentzian contribution), as determined by analyzing the Au 4f region of an Au standard. The fwhm and the relative binding energy positions of individual components were constrained to the same value in all XPS spectra of a given electronic region.

Cu A nanoparticles were supported on a Si(111) substrate by drop casting. After this, XPS measurements were conducted at beamline 9.3.2 of the Advanced Light Source, Lawrence Berkeley National Laboratory, with a photon energy of 695 eV. The intensities of the long-scan spectrum at Cu 3p, O 1s, and C 1s electronic regions were used for determining the organic layer thickness over the Cu A nanoparticles with the SESSA software.

Reflection electron energy loss spectroscopy (REELS) was measured using a SAM PHI 590A equipment at Instituto de Fisica del Litoral (CONICET-UNL). The powder sample was pelletized and inserted into the vacuum chamber for the measurements. An electron beam of 100 eV kinetic energy was directed toward the sample with an incident angle normal to the surface. Sputtering with 1 keV Ar^+^ ions with a normal incident angle to the surface was used to remove the surface impurities. Cu MVV, O KLL, and C KLL Auger signals were taken every 30 min to check the content of impurities. Adequate and convergent results were obtained after 185 min of irradiation. An almost complete C removal was reached after the cleaning process.

X-ray absorption spectroscopy (XAS) measurements of the Cu nanoparticles were carried out at the BL16-NOTOS beamline at the ALBA Synchrotron. The measurements were conducted in the transmission mode at the Cu K edge (8979 eV). Ten milligrams of the nanoparticle powder was mixed with 40 mg of amorphous SiO_2_, and the mixture was pressed into uniform pellets with a 13 mm diameter. The XAS spectra were recorded at room temperature and ambient pressure, using a Si(111) double-crystal monochromator and three ionization chambers 20 cm long, the first one before the sample filled with 100% N_2_ (12.0% absorption at 8979 eV) and both, the one after the sample and before the Cu foil reference used for energy calibration and the one after the reference filled with 40% N_2_ and 60% Ar (84.5% absorption at 8979 eV). Each XAS spectrum was derived from the average of at least 10 scans, with each scan taking roughly 6 min. Linear combination analysis was used for analyzing X-ray absorption near edge structure (XANES) regions using Cu(0) and Cu_2_O standards. The following equation was used for this linear combination:

7 $$\mu_{\exp}=C_{1}.\mu_{Cu\left(0\right)}+C_{2}.\mu_{Cu2O}$$

where μ_exp_ stands for the XANES spectrum of interest, while μ_Cu(0)_ and μ_Cu_2_O_ represent the XANES spectra of the Cu(0) and Cu_2_O standards, respectively. The coefficients were constrained to the condition of *C*_1_ + *C*_2_ = 1, and each individual coefficient was restricted to the range between 0 and 1. The ATHENA program was used for the deconvolution procedure.

The extended X-ray absorption fine structure (EXAFS) regions of the XAS data were analyzed following the standard procedure of data reduction^48^ using IFEFFIT.^49^ The EXAFS signal χ­(*k*) was extracted and then Fourier transformed using a Hanning window with a Δ*k* range of 9.0 Å^–1^. All data were *k* ^2^-weighted. FEFF6 was used to obtain the phase shift and amplitudes.^50^ The phase shifts and amplitudes were obtained by using Cu(0) and Cu_2_O clusters with crystal structures determined from the XRD data. The FT was adjusted up to the second shell around the absorbing atom. Only single scattering events were considered in the fitting process. The amplitude reduction factor, *S*_0_^2^, was fixed to 0.88, as determined from the fit of the Cu(0) foil standard. The resulting R-factor was around 0.005 for the different samples, indicating excellent agreement between the experimental data and the proposed model.

Molecular dynamics simulations were conducted in the LAMMPS package^51^ with the ReaxFF force field^52^ and a parametrization developed for the Cu/O systems^53^ to analyze Cu(0) and Cu_2_O interfaces at the atomistic level. Different configurations were analyzed, as shown in Figure S10 of the SI. Each proposed configuration was submitted to a thermal equilibration at 300 K and then cooled to 0.1 K to compute the desired properties (lattice parameters and radial distribution functions). The temperature and pressure along the periodic directions were controlled using the Nosé–Hoover thermostat and barostat (time constants of 100 and 1000 fs, respectively), and timesteps of 1.0 fs were employed. The radial distribution functions (RDFs) based on the Cu–O interatomic distances for individual phases (oxide and metal) in each system were computed using time averages over several configurations sampled at the final steps of thermal equilibration.

## Supplementary Material



## Notes

XRD, FTIR, XPS, HRTEM, XANES, and MD simulations complementary to the results presented in the main text. A table with the parameters obtained from the XRD and EXAFS analysis (PDF)
