# Supplementary material for: Local Strain Tuning in Cu Nanoparticles through Glucose-Mediated Synthesis
Source: ACS Omega. 2025 Oct 3;10(40):46624–33. doi: 10.1021/acsomega.5c03609 (PMC12529391; doi:10.1021/acsomega.5c03609)
Supplement: Supplementary file 1 [file ao5c03609_si_001.pdf]

Supporting Information

Local strain tuning in Cu nanoparticles through glucose-mediated synthesis

*Gustavo Z. Girotto, Kaue G. G. dos Santos, Ruan M. Martins, Marco A. H. Vogt, Silvia Montoro, Fernando Bonetto, Carlos Escudero, André R. Muniz, Fabiano Bernardi**

G. Z. Girotto, K. G. G. dos Santos, M. A. H. Vogt, F. Bernardi: Programa de Pós-Graduação em Física, Instituto de Física, Universidade Federal do Rio Grande do Sul (UFRGS). Av. Bento Gonçalves, 9500, Agronomia, Porto Alegre, Brazil.

R. M. Martins, A. R. Muniz: Departamento de Engenharia Química. Universidade Federal do Rio Grande do Sul (UFRGS). Rua Engenheiro Luiz Englert, s/nº – Prédio 12.204, Farroupilha, Porto Alegre, Brazil

S. Montoro, F. Bonetto: Instituto de Física del Litoral, CONICET-UNL. Guemes 3450, S3000GLN, Santa Fe, Argentina

F. Bonetto: Institute of Environmental Technology, CEET, VSB - Technical University of Ostrava, 17. listopadu 15/2172, Ostrava-Poruba, 70800, Czech Republic

C. Escudero: ALBA Synchrotron Light Source, Cerdanyola del Vallès, 08290 Barcelona, Spain

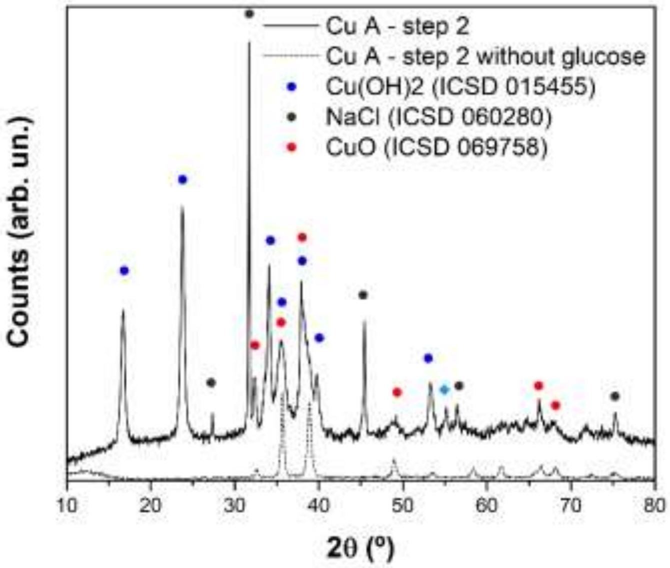

Figure S1. XRD pattern of Cu A sample during the beginning of step 2 of synthesis procedure and at the same stage without the addition of glucose.

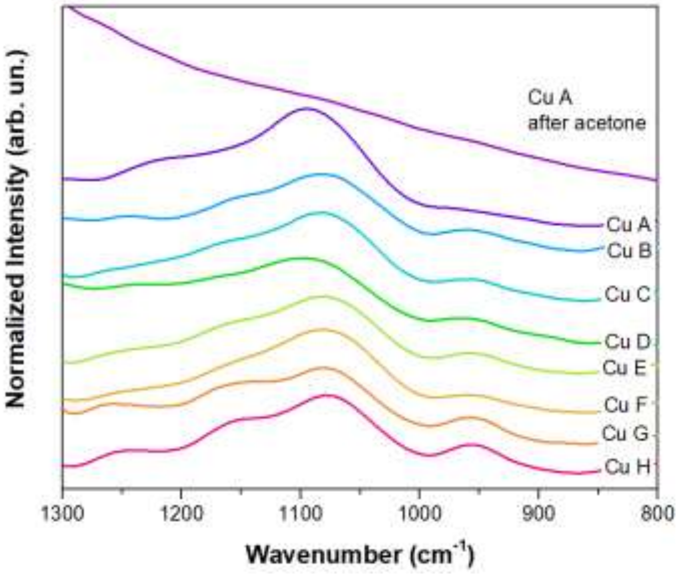

Figure S2. FTIR measurements of the samples in powder form after synthesis procedure.

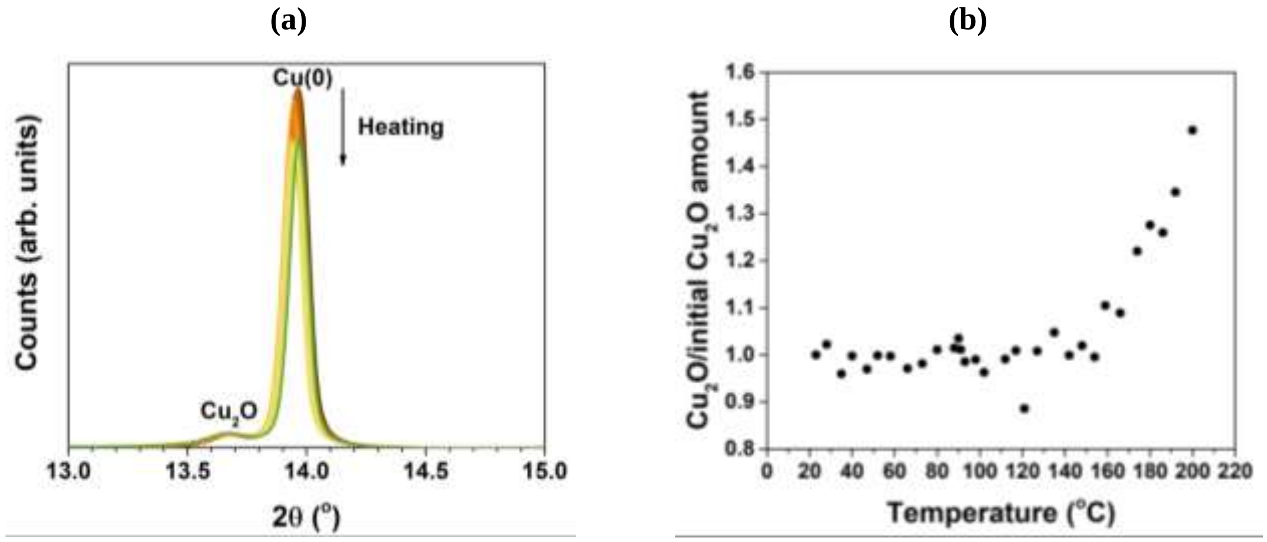

Figure S3. (a) In situ time-resolved XRD measurement of sample A during heating to 200 ^{o}C under 20% O_2 + 80% N_2 atmosphere. (b) Ratio between the area of the main Cu_2O Bragg reflection to its initial area value.

Table S1. Crystallite size obtained from XRD analysis.

Sample	Cu_2O (nm)	Cu(0) (nm)
Cu A	30 ± 5	30 ± 10
Cu B	29 ± 5	30 ± 10
Cu C	27 ± 10	30 ± 10
Cu D	100 ± 30	40 ± 10
Cu E	70 ± 20	21 ± 8
Cu F	15 ± 4	-
Cu G	40 ± 10	-
Cu H	110 ± 30	-

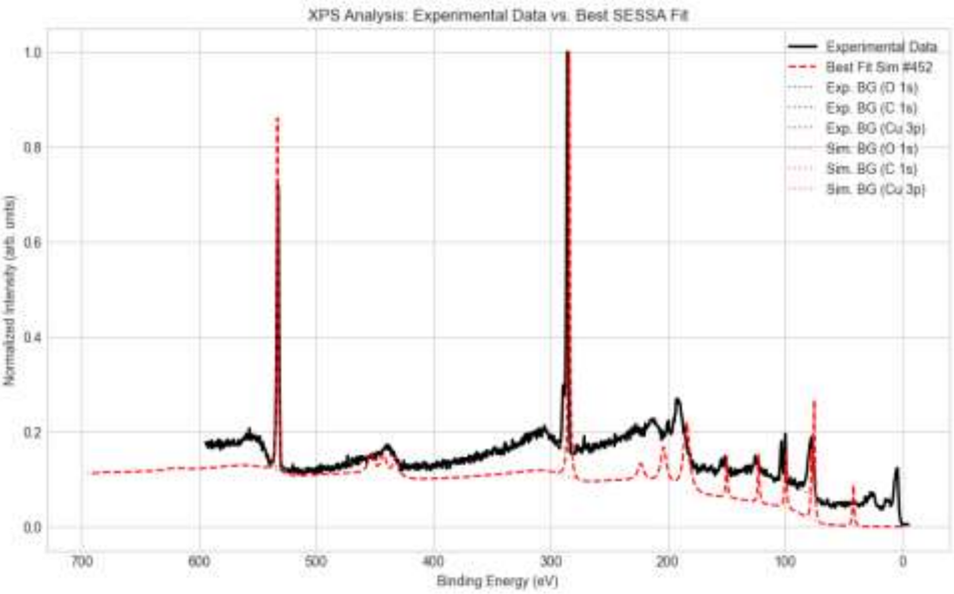

Figure S4. Long scan XPS spectrum along with the fit result from SESSA software.

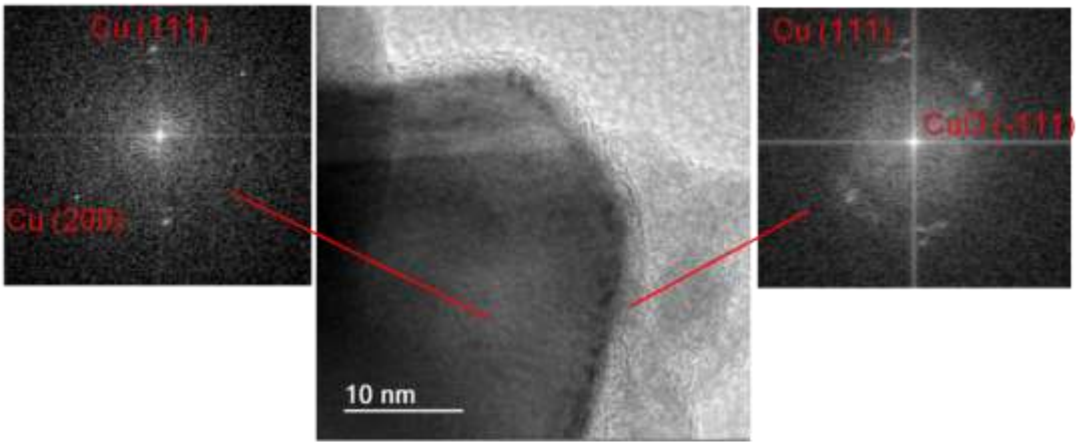

Figure S5. HRTEM image of Cu A sample and its respective FFT at the core and shell regions.

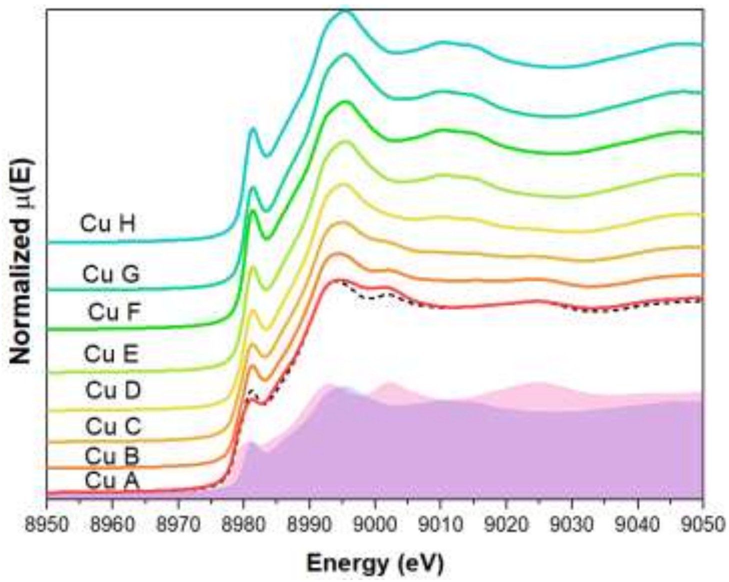

Figure S6. XANES measurements at the Cu K edge of the Cu nanoparticles synthesized. The XANES spectrum of the Cu A sample is shown together with the fit result (dashed line) and the Cu(0) (pink filled area) and Cu_2O (violet dashed area) standards.

Table S2. Parameters obtained from the fit of the EXAFS measurements at Cu K edge.

			Cu-O from Cu_2O			Cu-Cu from Cu(0)			Cu-Cu from Cu_2O			Cu-O from Cu_2O		
**Sample**	**% Cu(0) XANES**	**%Cu_2O XANES**	**N**	**R (Å)**	**σ(Å^2)**	**N**	**R (Å)**	**σ(Å^2)**	**N**	**R (Å)**	**σ(Å^2)**	**N**	**R (Å)**	**σ(Å^2)**
Cu A	0.54 (0.06)	0.46 (0.06)	1.1 (0.4)	1.89 (0.04)	0.006 (0.002)	5.4 (0.2)	2.543 (0.001)	0.008 (0.003)	2 (1)	3.05 (0.07)	0.02 (0.009)	4 (2)	3.4 (0.1)	0.02 (0.01)
Cu B	0.45 (0.07)	0.55 (0.07)	1.2 (0.3)	1.87 (0.02)	0.005 (0.002)	5.2 (0.2)	2.548 (0.003)	0.008 (0.004)	7 (1)	3.06 (0.06)	0.025 (0.008)	6 (2)	3.4 (0.1)	0.02 (0.01)
Cu C	0.34 (0.07)	0.66 (0.07)	1.6 (0.3)	1.87 (0.02)	0.006 (0.002)	4.2 (0.3)	2.550 (0.005)	0.009 (0.003)	5.1 (0.8)	3.03 (0.07)	0.024 (0.008)	7 (2)	3.4 (0.1)	0.03 (0.01)
Cu D	0.18 (0.04)	0.82 (0.04)	2.0 (0.2)	1.86 (0.01)	0.006 (0.002)	2.6 (0.5)	2.548 (0.007)	0.009 (0.006)	3.9 (0.4)	2.99 (0.05)	0.017 (0.006)	5 (1)	3.47 (0.07)	0.016 (0.009)
Cu E	0.04 (0.05)	0.96 (0.05)	2.4 (0.1)	1.86 (0.01)	0.006 (0.002)	0.9 (0.8)	2.552 (0.009)	0.009 (0.007)	6 (0.3)	2.99 (0.08)	0.019 (0.006)	4 (1)	3.48 (0.08)	0.012 (0.007)
Cu F	0.04 (0.07)	0.96 (0.04)	2.1 (0.1)	1.852 (0.004)	0.004 (0.001)	0.3	2.556	0.023	8.1 (0.3)	3.00 (0.05)	0.021	6.6 (0.7)	3.51 (0.09)	0.027 (0.009)
Cu G	0	1 (0.03)	2.6 (0.2)	1.86 (0.01)	0.006 (0.002)	--	--	--	6.5 (0.3)	2.98 (0.06)	0.018	3.3 (0.6)	3.49 (0.08)	0.011 (0.008)
Cu H	0	1 (0.03)	2.3 (0.1)	1.853 (0.002)	0.005 (0.002)	--	--	--	7.4	2.99 (0.05)	0.02	5.4 (0.7)	3.49 (0.08)	0.019 (0.009)

Figure S7(a) shows the HR-TEM of a selected region in the Cu A sample, and S7(b) shows the respective FFT, with bright spots indicating 0.31 nm/c features. A line profile was extracted through a rectangular selection in (a), averaging along the width of the ROI. The result is displayed in Figure S7(c). The data was then trimmed 10 points in the low-end region and 25 points in the high-end region to avoid spurious artifacts. The data was cleaned of noise and low-frequency modulation through a Hilbert transform to calculate the signal envelope, which is smoothed through a Savitsky-Golay filter using 11 points per window and a 3rd-order polynomial. The data is normalized by this envelope and

subtracted by another 3rd-order polynomial baseline. Finally, a bandpass Butterworth filter is used to isolate the frequency spacing of interest detected previously with a 2D FFT of around 0.3 nm^{-1}, using a fractional bandwidth of 0.5, the final result of which is shown in S7(d). Figure S7(e) shows the complex Continuous Wavelet Transform with a Morlet function applied between frequencies of 2 nm^{-1} and 10 nm^{-1} (below the Nyquist frequency of ~19 nm^{-1}). Strong features appear in two different regions, below 9 nm and above 12 nm on the z-axis. The difference in overall intensity is affected by the thickness of the crystal that turns thinner near the interface. There is a drop between ~9 and ~12 nm that indicates an interface, grain boundary, or large defect. Although the exact shape of the wavelet is determined by the procedure adopted, the fluctuations in the frequency axis represent a local strain inside the grains. The strain obtained from this analysis is around 1%, which is consistent with that obtained from EXAFS analysis (2%).

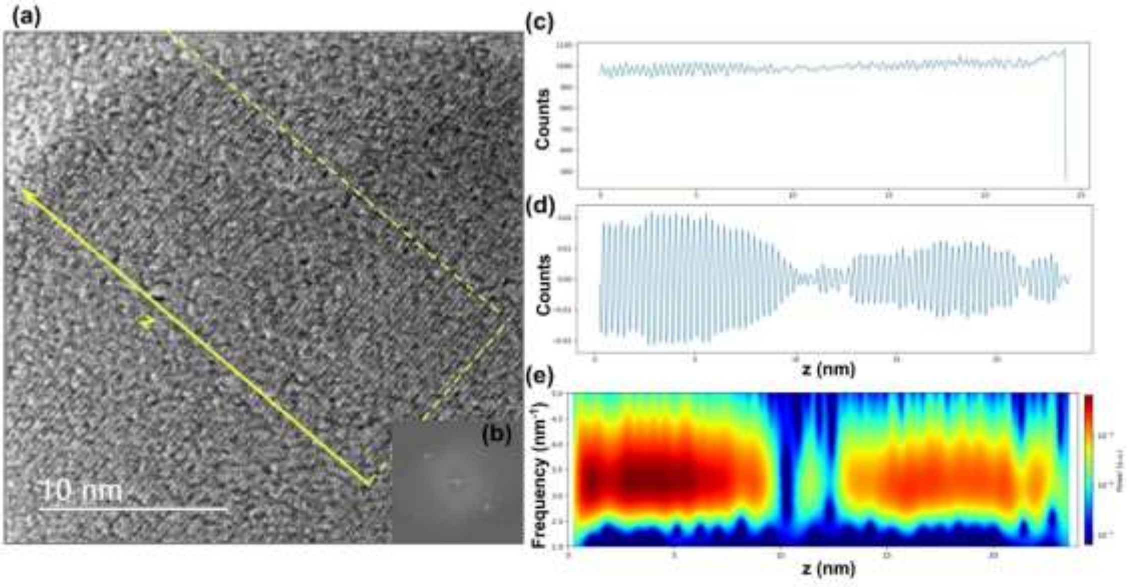

Figure S7. (a) HRTEM image with (b) the FFT of the highlighted region in the inset, (c) line profiling from the rectangular selection in (a) and (d) the profiling after a bandpass filter, and (e) complex continuous wavelet transform.

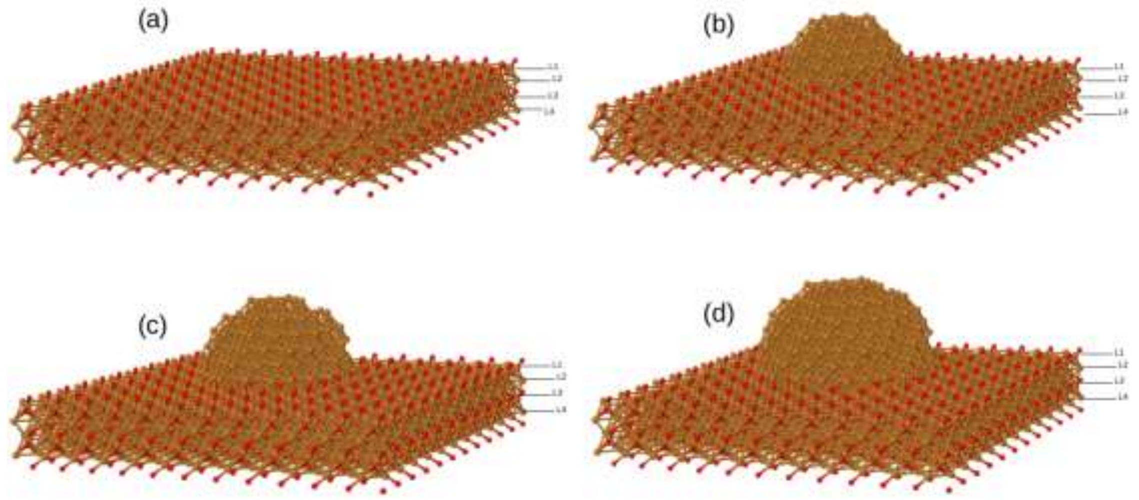

Figure S8. Initial configurations for the (a) Cu_2O pristine slab and slab/nanoparticle systems with nanoparticles of (b) 1.5 nm, (c) 1.8 nm, and (d) 2.2 nm.

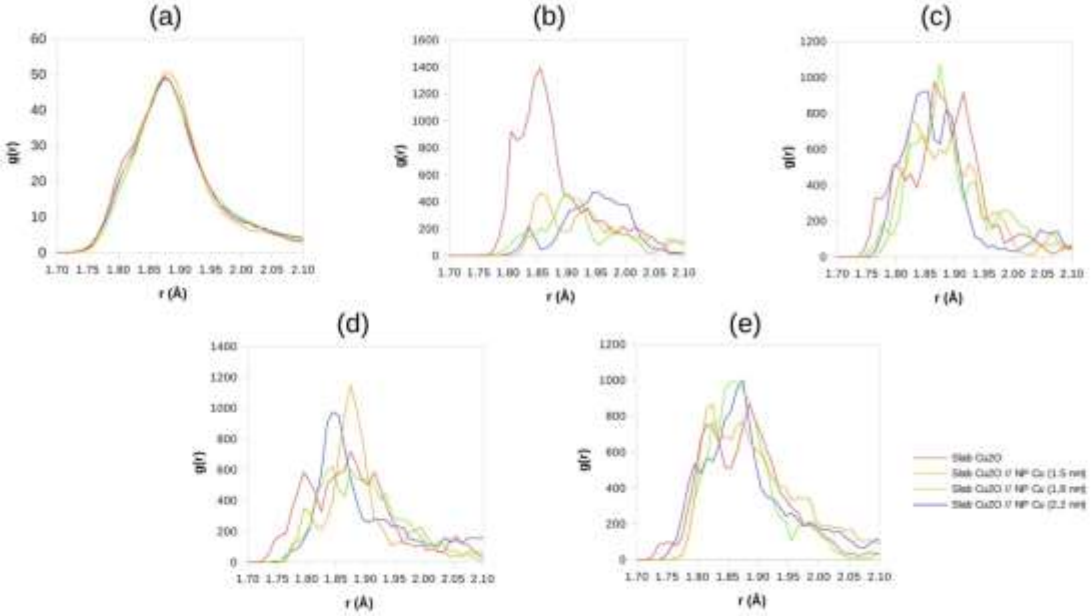

Figure S9. First peak of the RDFs curves based on the Cu-O interatomic distances for (a) the full systems, and for the (b) first (L1), (c) second (L2), (d) third (L3), and (e) fourth (L4) layers of the Cu_2O substrate.

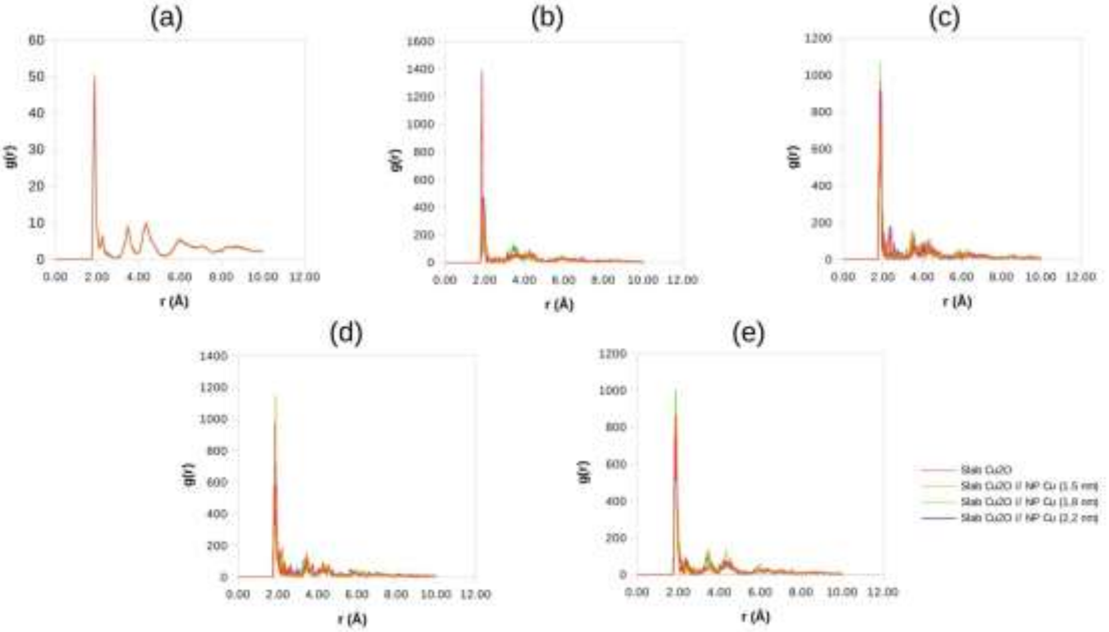

Figure S10. Full RDF curves based on the Cu-O interatomic distances for (a) the full systems, and for the (b) first (L1), (c) second (L2), (d) third (L3), and (e) fourth (L4) layers of the Cu_2O substrate.
